# Supplementary material for: What Does ‘Preconception Health’ Mean to People? A Public Consultation on Awareness and Use of Language
Source: Health Expect. 2024 Aug 24;27(4):e14181. doi: 10.1111/hex.14181 (PMC11344208; doi:10.1111/hex.14181)
Supplement: Supplementary file 1 — Supporting information. [file HEX-27-e14181-s001.docx]

**Supplementary File 1**

**1) Anonymous evolution poll results from session 1, N = 54 public contributors**


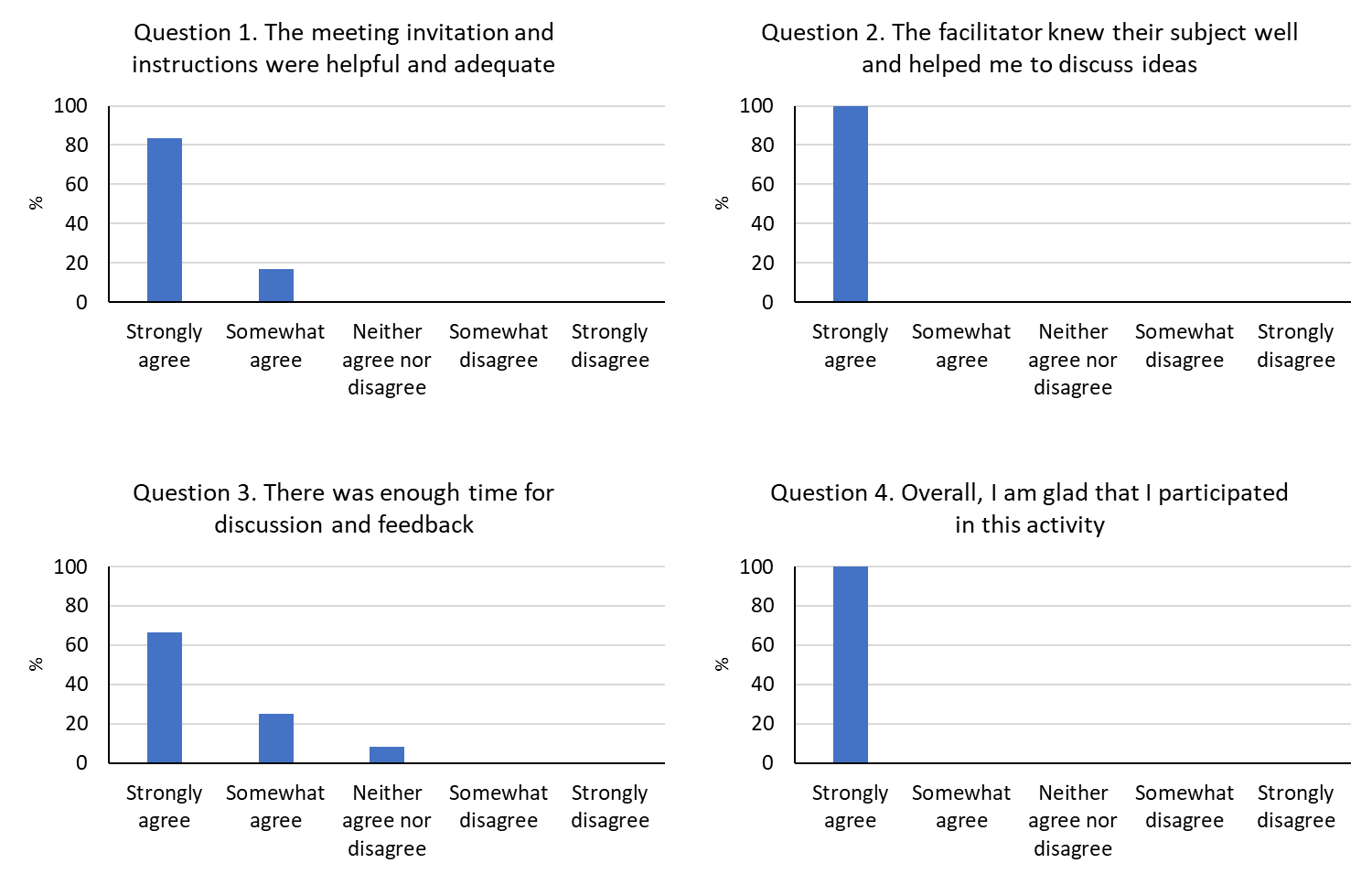


**2) Anonymous Feedback Form results from session 1, N = 32 public contributors**

Selected quotes to cover common themes:

Please provide any feedback about what you enjoyed or found useful about this activity.

“There was no pressure and the conversation flowed well. Everyone got a chance to say something and the facilitator was very knowledgeable and engaging and made me feel like my contribution was valuable.”

“I was really intrigued on the subject. I hadn't realised that our health from many years before pregnancy would make an impact on the ability to get pregnancy, and more so the complications during childbirth and pregnancy.”

“Great to meet everyone, there was so much to learn from other people’s experiences and thoughts.”

“Among everyone I don’t think there was a master on the subject, but they all made salient points. I really enjoyed the discussions as they provided me with things I didn’t know before!”

Is there anything you think could be improved?

“A little more time would have been good.”

“Might have been useful to have some examples of the key messages as they stand.”

“Specific examples of wording that is currently being used and the context it's used in, as well as examples of potential other wording.”

“There was some repetition as some questions were asked multiple times. Although I guess this was to involve everyone in the discussion, those who hadn’t spoken yet.”

Is there anything that you will take away as a result of the discussion?

“Made me think about my own preconception health, and the effects my choices will have on my body later in life.”

“I now have a different perspective to the importance of health before having children and the discussion has made me recognise the lack of education in this area.”

“I learned that the health of the male before pregnancy can impact the health of the baby too! More information on this is needed.”

“The discussion on positive and motivational approaches to promotion of health before pregnancy was really interesting. Also, the importance of language used in messages and how these may affect information uptake.”

Please use the space below to comment on any other part of the activity.

“Thank you so much for the opportunity to be involved in such an interesting discussion! I plan to spend the voucher on some books on health to keep learning, so thank you so much!”

“Really interesting! Thanks! Looking forward to being further involved.”
